# Usefulness of ECG to differentiate apical hypertrophic cardiomyopathy from non-ST elevation acute coronary syndrome

**DOI:** 10.1186/s12872-020-01592-0

**Published:** 2020-06-23

**Authors:** Yirao Tao, Jing Xu, Samira Yerima Bako, Xiaobo Yao, Donghui Yang

**Affiliations:** 1 Department of Cardiology, The Second Affiliated Hospital of Dalian Medical University, Dalian, Liaoning China; 2 Department of Cardiology, Shanghai East Hospital, Shanghai Tongji University School of Medicine, Shanghai, China

**Keywords:** Apical hypertrophic cardiomyopathy, Non-ST elevation acute coronary syndrome, Electrocardiogram

## Abstract

**Background:**

Apical hypertrophic cardiomyopathy (ApHCM) is a phenotypic variant of nonobstructive HCM. ApHCM is characterized by left ventricular hypertrophy involve the distal apex. The electrocardiographic character of ApHCM can mimic non-ST elevation acute coronary syndrome (NSTEACS), triggering a series of studies and treatments that may be unnecessary. This study aimed to clarify the electrocardiogram (ECG) differences between the two diseases.

**Methods:**

Initial ECG recordings of 41 patients with ApHCM and 72 patients with NSTEACS were analyzed retrospectively. We analyzed the voltage of negative T (neg T) and R wave, the change of ST-segment as well as the number of leads with neg T wave in the 12-lead ECGs.

**Results:**

Across the 12-lead ECGs, the magnitude of R wave significantly differed between ApHCM and NSTEACS in 10 leads excluding leads aVR and V1. ApHCM was associated with a greater maximal amplitude of R wave in lead V5 (3.13 ± 1.08 vs. 1.38 ± 0.73 mV, *P* <  0.001). The magnitude of T wave significantly differed between ApHCM and NSTEACS in 10 leads excluding leads II and V1. ApHCM was associated with a greater maximal amplitude of neg T wave in lead V4 (0.85 ± 0.69 vs. 0.35 ± 0.23 mV, *P* <  0.001). The frequency of giant neg T (1mv or more) wave was higher in ApHCM (36.5% vs. 0%, *P* <  0.001). The magnitude of ST-segment deviation significantly differed between ApHCM and NSTEACS in 10 leads excluding leads aVF and V2. ApHCM was associated with a greater maximal amplitude of ST-segment depression in lead V5 (0.19 ± 0.07 vs. 0.03 ± 0.06 mV, *P* <  0.001). The number of leads with neg T wave also differed between ApHCM and NSTEACS (6.75 ± 1.42 vs. 6.08 ± 1.51, *P* = 0.046). The sum of R wave in lead V5, neg T wave in lead V6 and ST-segment depression in lead V4 > 2.585 mV identified ApHCM with 90.2% sensibility and 87.5% specificity, representing the highest diagnostic accuracy.

**Conclusions:**

Compared with NSTEACS patients, ApHCM patients presented higher R and neg T wave voltage as well as a greater ST-segment depression in the 12-lead ECG. The ECG characteristics can help to differentiate ApHCM from NSTEACS in clinical setting.

## Background

Apical hypertrophic cardiomyopathy (ApHCM) is complex phenotypic variant of the classical hypertrophic cardiomyopathy [1]. It can be asymptomatic or present with dyspnea, chest pain, syncope as well as severe diastolic dysfunction even sudden cardiac death [2–4]. Given its diverse presentation forms, clinical evaluation alone cannot be relied upon. Understanding the unique electrocardiogram (ECG) features of ApHCM can be of assistance in the diagnostic process of this uncommon disease.

Because of similar clinical manifestations and large negative T (neg T) waves in precordial leads on ECG, most previous case reports misdiagnosed ApHCM as non-ST elevation acute coronary syndrome (NSTEACS) [5–11]. Differentiation of these two diseases can be challenging, but has an important role in the selection of an appropriate treatment strategy. The 12-lead ECG is the simplest and widely used clinical diagnostic test. Several ECG features of ApHCM have been reported which may help to make these distinctions [12, 13]. These include higher T wave voltage and peak voltage, T wave asymmetry and higher R waves. Nevertheless, previous studies were restricted to case reports or a small group of patients, the serial ECG differences between ApHCM and NSTEACS have not been sufficiently elucidated.

Our aim was to explore the ECG patterns in ApHCM patients and compare them with NSTEACS patients to distinguish between the two diagnoses, thereby helping choose a more appropriate treatment strategy and finally improving clinical outcome.

## Methods

### Participants

We retrospectively studied 113 consecutive patients (41 patients with ApHCM and 72 patients with NSTEACS) who were admitted to our Cardiology department within 7 days from symptom onset between April 2015 and April 2019. Exclusion criteria included ventricular pacing, atrial fibrillation or flutter and left or right bundle branch block. The basic data of gender, age, history of smoking and alcohol, diabetes and hypertension were also recorded. This study was approved by our Institutional Ethics Committee and all participants provided informed consent according to the Declaration of Helsinki.

### ApHCM group

Definition of ApHCM relies on demonstrating left ventricular hypertrophy predominating in the distal apex by cardiovascular magnetic resonance imaging or transthoracic echocardiography, with a wall thickness ≥ 15 mm of the apex and maximal apical/posterior wall ratio ≥ 1.5 [14, 15].

### NSTEACS group

NSTEACS group included subjects with unstable angina (UA) and acute non-ST segment elevation myocardial infarction (NSTEMI) [16]. All patients presented with precordial T waves inversion on admission ECG and have an ischemic symptom, such as new-onset, rest, or increasing angina. Patients with left ventricular hypertrophy diagnosed by cardiovascular magnetic resonance or transthoracic echocardiography were excluded from this group.

### ECG evaluation

A standard 12-lead ECG on admission was recorded at a 10 mm/mV amplitude and a 25 mm/s speed. QT interval was corrected using the Bazett formula [17]. The ST-segment deviation was measured manually 0.08 s after the J-point in each lead [18]. We analyzed the following ECG differences: (1) R wave amplitude in 12 leads; (2) T wave amplitude in 12 leads; (3) amplitude of ST-segment deviation in 12 leads; (4) giant neg T wave (1 mV or morein any ECG lead [19]); (5) the number of leads with neg T wave; (6) total amplitude of neg T waves. All ECGs were measured by a single investigator who was blinded to clinical information. The average values came from three continuous sinus beat.

### Statistics

Continuous data were described as mean (± SD) and compared by Mann-Whitney U-test or Student’s T-test. Categorical data are described as numbers and percentages and compared by Fisher’s exact test or Chi-square test. Youden’s index which derived from receiver operator characteristic (ROC) curves evaluated the cut-off value, while the area under curve (AUC) evaluated which ECG marker represented the highest diagnostic accuracy. *P*-value < 0.05 was considered significant. SPSS, version 22.0 software was used to manage the data.

## Results

### Study group

The baseline characteristics were presented in Table 1. The mean (± SD) age was 69.55 (±10.75) years. 46.9% of participants were men. Patients with ApHCM were more likely to be male and had a larger left atrium, higher left ventricular ejection fraction (LVEF), thicker left ventricular posterior wall (LVPW) and interventricular septal (IVS) as well as a lower prevalence of diabetes mellitus than those in NSTEACS group. Other characteristics did not differ between ApHCM and NSTEACS.

**Table 1:** Baseline Characteristics

	ApHCM (*n* = 41)	NSTEACS (*n* = 72)	*P*-value
Men	27 (65.9)	26 (36.1)	0.002
Age (years)	68.29 ± 11.12	70.27 ± 10.55	0.384
Smoking	12 (29.3)	22 (30.6)	0.886
Drinking	5 (12.2)	7 (9.7)	0.682
Hypertension	28 (68.3)	54 (75)	0.442
Diabetes mellitus	5 (12.2)	21 (29.2)	0.039
LAD (mm)	42.09 ± 4.54	39.38 ± 4.71	0.004
LVEDd (mm)	47.82 ± 3.93	47.40 ± 5.58	0.320
LVEDs (mm)	29.97 ± 3.01	32.08 ± 5.85	0.158
IVS (mm)	11.65 ± 2.42	9.61 ± 1.21	< 0.001
LVPW (mm)	9.82 ± 1.04	9.25 ± 0.91	0.001
LVEF (%)	65.65 ± 4.89	59.13 ± 9.32	< 0.001
E/A	0.97 ± 0.59	0.84 ± 0.37	0.853
E/Em	10.12 ± 5.92	10.10 ± 4.97	0.795

*Abbreviation*: *A* Late diastolic inflow velocity, *ApHCM* Apical hypertrophic cardiomyopathy, *E* Early diastolic inflow velocity, *Em* Early diastolic annular tissue velocity, *IVS* Interventricular septal, *LAD* Left atrial diameter, *LVEDd* Left ventricular end diastolic diameter, *LVEDs* Left ventricular end systolic diameter, *LVEF* LEFT ventricular ejection fraction, *LVPW* Left ventricular posterior wall, *NSTEACS* Non-ST elevation acute coronary syndrome

### ECG findings

**Fig. 1:**
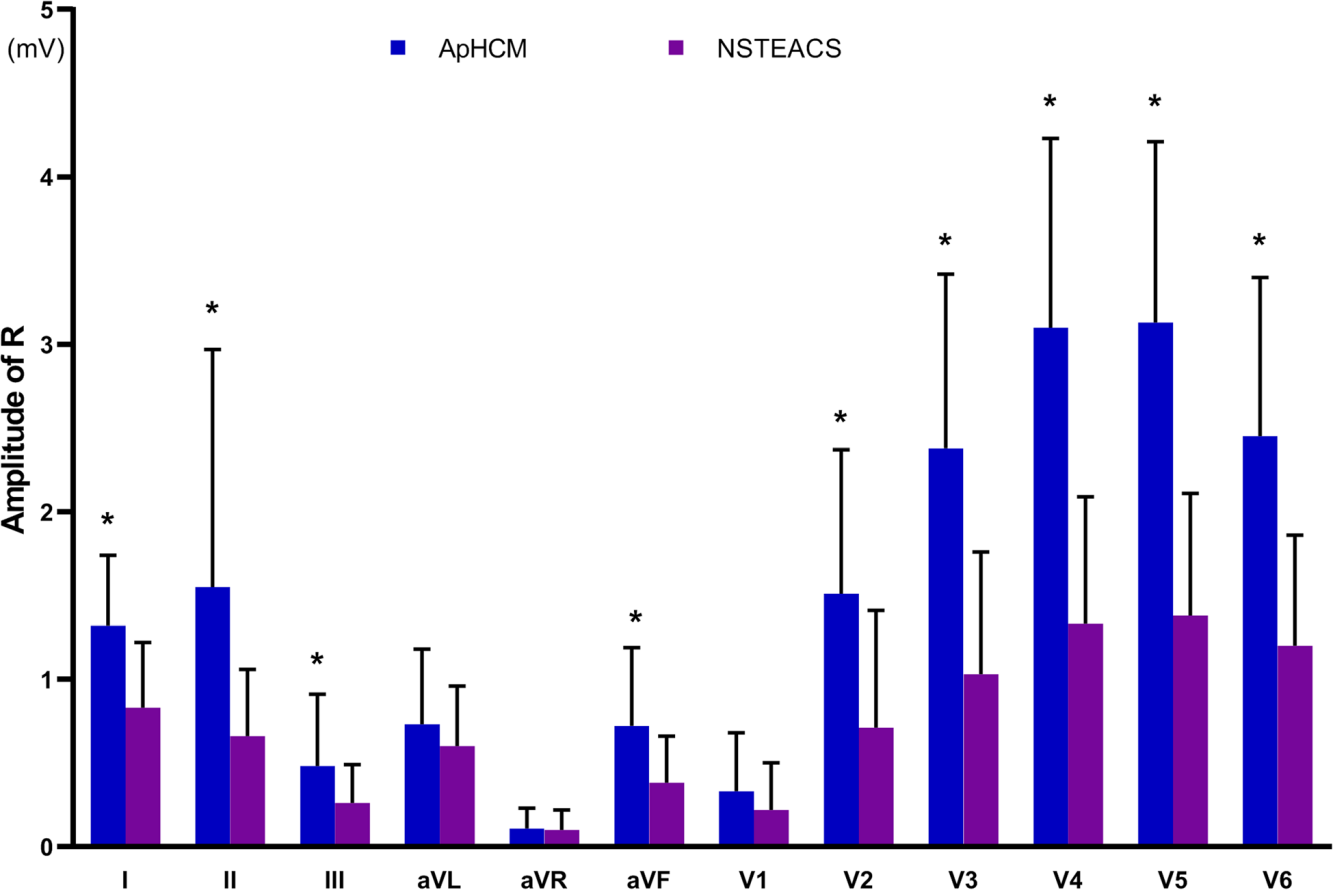
Comparison of R waves amplitude between apical hypertrophic cardiomyopathy (ApHCM) and non-ST elevation acute coronary syndrome (NSTEACS). *P < 0.05 vs. NSTEACS

**Fig. 2:**
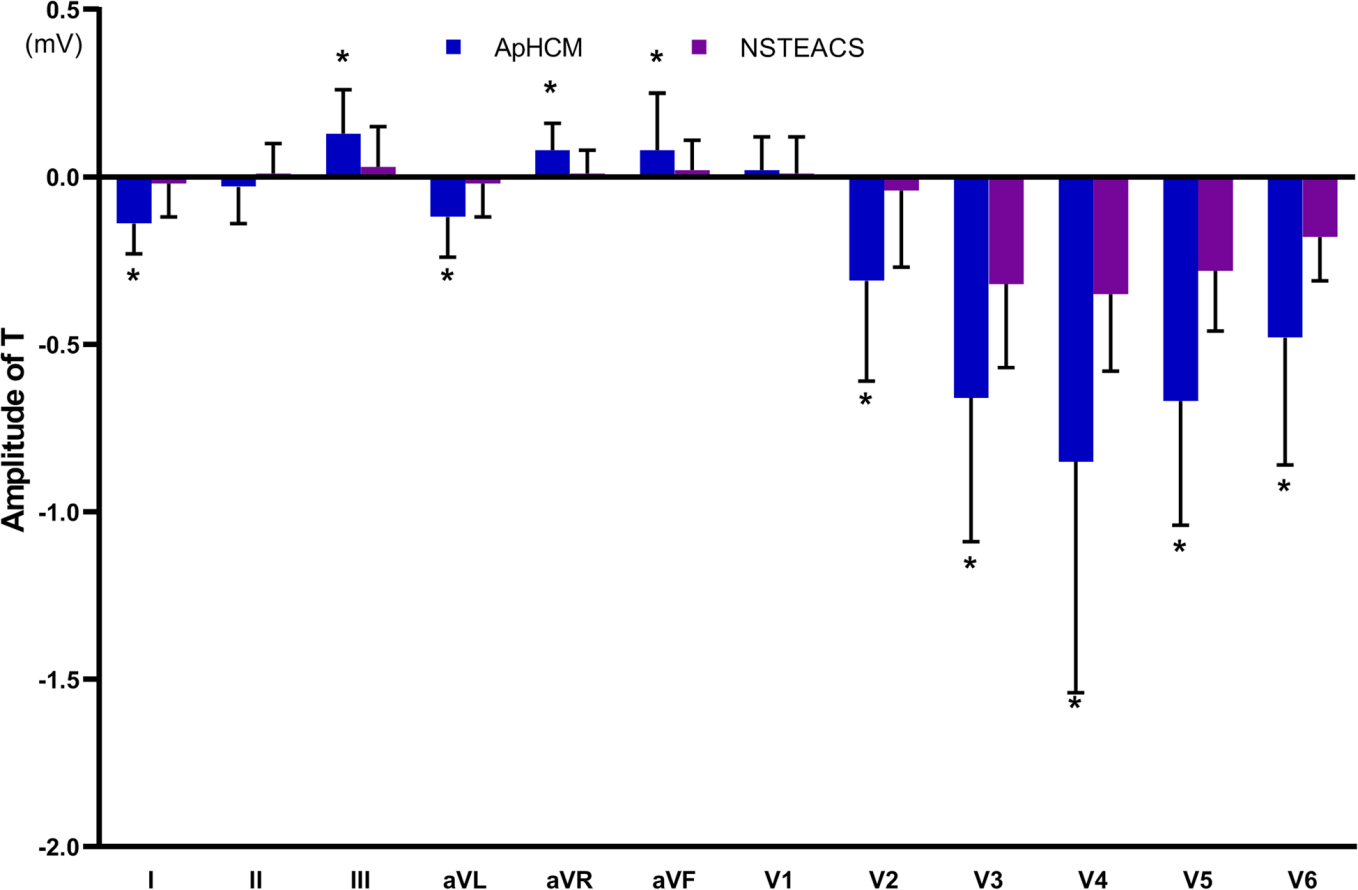
Comparison of T waves amplitude between apical hypertrophic cardiomyopathy (ApHCM) and non-ST elevation acute coronary syndrome (NSTEACS). *P < 0.05 vs. NSTEACS

**Fig. 3:**
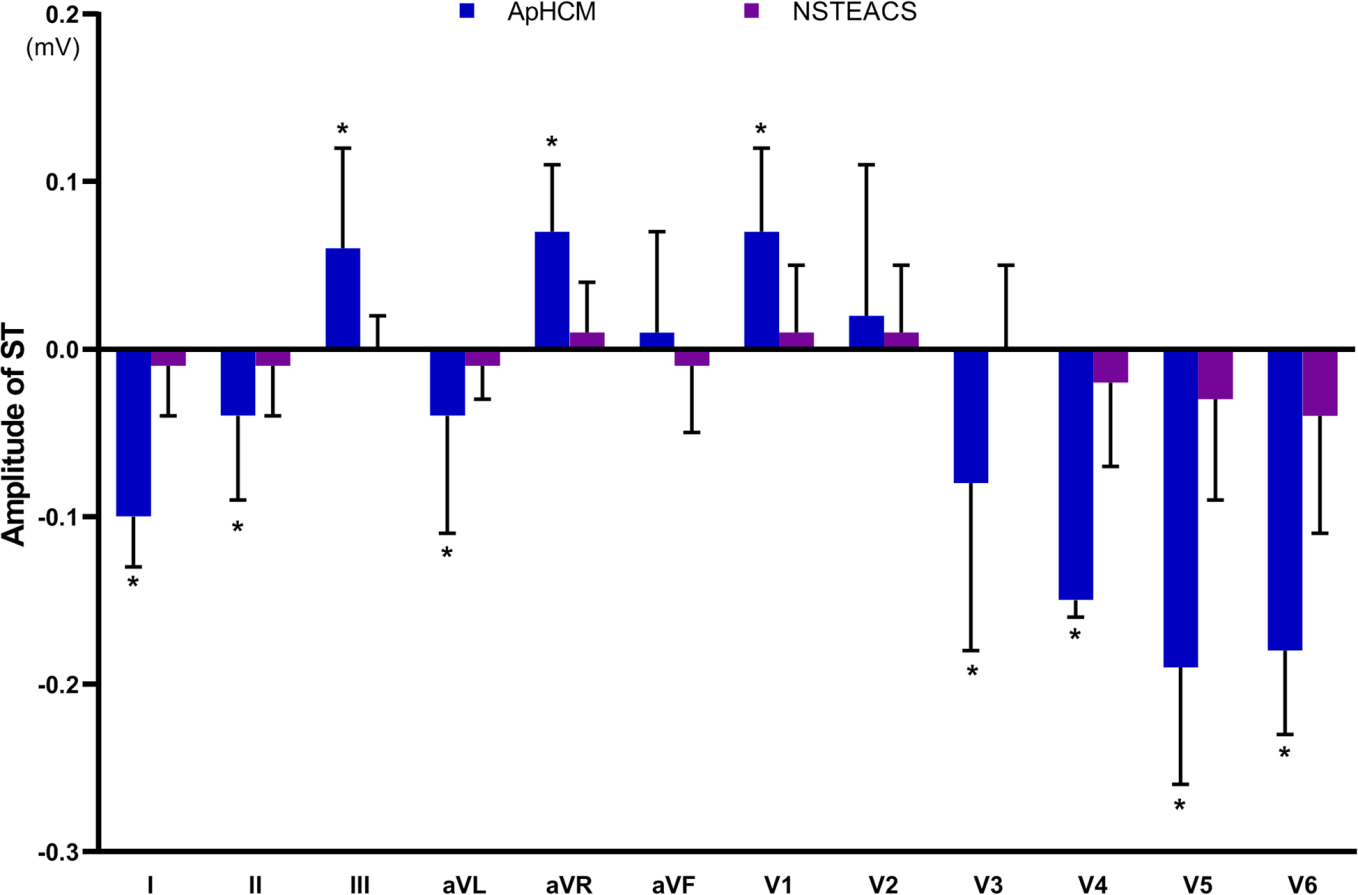
Comparison of ST-segments deviation between apical hypertrophic cardiomyopathy (ApHCM) and non-ST elevation acute coronary syndrome (NSTEACS). *P < 0.05 vs. NSTEACS

QRS interval and QTc interval did not differ significantly between ApHCM and NSTEACS. The magnitude of R wave significantly differed between ApHCM and NSTEACS in 10 leads, excluding leads aVR and V1. ApHCM was associated with a greater maximal amplitude of R wave (3.13 ± 1.08 vs. 1.38 ± 0.73 mV, *P* <  0.001) in lead V5. The comparison of QRS interval, QTc interval and R wave were shown in Table 2 and Fig. 1. Neg T waves were consistently observed in leads I, aVL and V2-V6 in ApHCM. The magnitude of T wave significantly differed between ApHCM and NSTEACS in 10 leads, excluding leads II and V1. ApHCM was associated with a greater maximal amplitude of neg T wave (0.85 ± 0.69 vs. 0.35 ± 0.23 mV, *P* <  0.001) in lead V4. The frequency of giant neg T wave was higher in ApHCM (36.5% vs. 0%, *P* <  0.001). Besides, a greater total magnitude of neg T waves (3.38 ± 1.75 vs. 1.47 ± 0.85 mV, *P* <  0.001) and a larger number of leads with neg T wave (6.75 ± 1.42 vs. 6.08 ± 1.51, *P* = 0.046) were found in ApHCM. The comparisons of T wave were shown in Table 3 and Fig. 2. ST-segments elevation in leads aVR, V1 and ST-segments depression in leads I, V4-V6 were consistently observed in ApHCM. The magnitude of ST-segment deviation significantly differed between ApHCM and NSTEACS in 10 leads, excluding leads aVF and V2. ApHCM was associated with a greater maximal amplitude of ST-segment depression (0.19 ± 0.07 vs. 0.03 ± 0.06 mV, *P* <  0.001) in lead V5. The comparisons of ST-segment deviation in 12 leads were shown in Table 4 and Fig. 3.

**Table 2:** The comparison of QRS interval, QTc interval and R wave between ApHCM and NSTEACS in ECG

	ApHCM (*n* = 41)	NSTEACS (*n* = 72)	*P*-value
QRS interval (ms)	97.26 ± 11.20	95.69 ± 14.54	0.174
QTc interval (ms)	441.24 ± 26.34	438.75 ± 43.53	0.254
R wave in I (mV)	1.31 ± 0.42	0.83 ± 0.39	< 0.001
R wave in II (mV)	1.55 ± 1.42	0.66 ± 0.40	< 0.001
R wave in III (mV)	0.48 ± 0.43	0.26 ± 0.23	0.004
R wave in aVR (mV)	0.11 ± 0.12	0.10 ± 0.12	0.299
R wave in aVL (mV)	0.73 ± 0.45	0.60 ± 0.36	0.121
R wave in aVF (mV)	0.72 ± 0.47	0.38 ± 0.28	< 0.001
R wave in V1 (mV)	0.33 ± 0.35	0.22 ± 0.28	0.029
R wave in V2 (mV)	1.51 ± 0.86	0.71 ± 0.70	< 0.001
R wave in V3 (mV)	2.38 ± 1.04	1.03 ± 0.73	< 0.001
R wave in V4 (mV)	3.10 ± 1.13	1.33 ± 0.76	< 0.001
R wave in V5 (mV)	3.13 ± 1.08	1.38 ± 0.73	< 0.001
R wave in V6 (mV)	2.45 ± 0.95	1.20 ± 0.66	< 0.001

*Abbreviation*: *ApHCM* Apical hypertrophic cardiomyopathy, *NSTEACS* Non-ST elevation acute coronary syndrome

**Table 3:** The comparison of T wave between ApHCM and NSTEACS in ECG

	ApHCM (*n* = 41)	NSTEACS (*n* = 72)	*P*-value
T wave in I (mV)	−0.14 ± 0.09	− 0.02 ± 0.10	< 0.001
T wave in II (mV)	−0.03 ± 0.11	0.01 ± 0.09	0.070
T wave in III (mV)	0.13 ± 0.13	0.03 ± 0.12	< 0.001
T wave in aVR (mV)	0.08 ± 0.08	0.01 ± 0.07	< 0.001
T wave in aVL (mV)	−0.12 ± 0.12	− 0.02 ± 0.10	< 0.001
T wave in aVF (mV)	0.08 ± 0.17	0.02 ± 0.09	0.002
T wave in V1 (mV)	0.02 ± 0.10	0.01 ± 0.11	0.230
T wave in V2 (mV)	−0.31 ± 0.30	− 0.04 ± 0.23	< 0.001
T wave in V3 (mV)	−0.66 ± 0.43	−0.32 ± 0.25	< 0.001
T wave in V4 (mV)	−0.85 ± 0.69	− 0.35 ± 0.23	< 0.001
T wave in V5 (mV)	−0.67 ± 0.37	− 0.28 ± 0.18	< 0.001
T wave in V6 (mV)	− 0.48 ± 0.38	− 0.18 ± 0.13	< 0.001
Giant neg T wave (number of cases/%)	15 (36.5)	0	< 0.001
Number of leads with neg T wave	6.75 ± 1.42	6.08 ± 1.51	0.046
Total amplitude of neg T waves (mV)	3.38 ± 1.75	1.47 ± 0.85	< 0.001

*Abbreviation*: *ApHCM* Apical hypertrophic cardiomyopathy, *NSTEACS* Non-ST elevation acute coronary syndrome, *neg* Negative

**Table 4:** The comparison of ST-segment deviation in 12 leads between ApHCM and NSTEACS in ECG

	ApHCM (*n* = 41)	NSTEACS (*n* = 72)	*P*-value
ST-segment in I (mV)	−0.10 ± 0.03	−0.01 ± 0.03	< 0.001
ST-segment in II (mV)	−0.04 ± 0.05	−0.01 ± 0.03	0.002
ST-segment in III (mV)	0.06 ± 0.06	0.00 ± 0.02	< 0.001
ST-segment in aVR (mV)	0.07 ± 0.04	0.01 ± 0.03	< 0.001
ST-segment in aVL (mV)	−0.04 ± 0.07	−0.01 ± 0.02	< 0.001
ST-segment in aVF (mV)	0.01 ± 0.06	−0.01 ± 0.04	0.057
ST-segment in V1 (mV)	0.07 ± 0.05	0.01 ± 0.04	< 0.001
ST-segment in V2 (mV)	0.02 ± 0.09	0.01 ± 0.04	0.054
ST-segment in V3 (mV)	−0.08 ± 0.10	0.00 ± 0.05	< 0.001
ST-segment in V4 (mV)	−0.15 ± 0.01	−0.02 ± 0.05	< 0.001
ST-segment in V5 (mV)	−0.19 ± 0.07	−0.03 ± 0.06	< 0.001
ST-segment in V6 (mV)	−0.18 ± 0.05	−0.04 ± 0.07	< 0.001

*Abbreviation*: *ApHCM* Apical hypertrophic cardiomyopathy, *NSTEACS* Non-ST elevation acute coronary syndrome

**Fig. 4:**
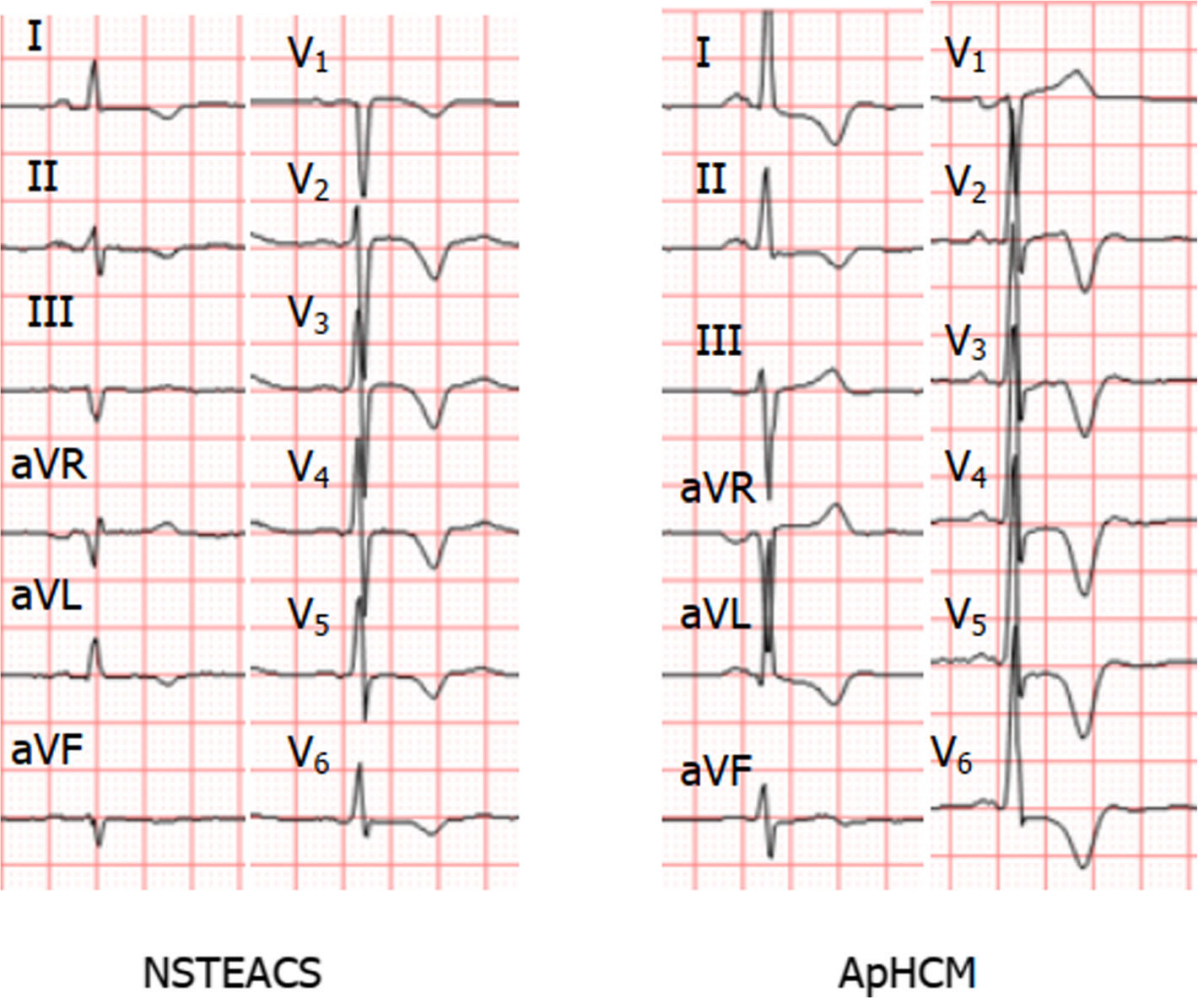
Representative ECGs of apical hypertrophic cardiomyopathy (ApHCM) and non-ST elevation acute coronary syndrome (NSTEACS). Left (NSTEACS): negative T waves were observed in leads I, II, aVL, V1-V6. Right (ApHCM): negative T waves were observed in leads I, II, aVL, V2-V6, ST-segment elevation in leads III, aVR, V1 and ST-segment depression in leads I, aVL, V4-V6

R wave in lead V5 represent the highest sensitivity (90.2%) for ApHCM compared with the other leads with the cut-off value of 2.07 mV.Neg T wave in lead V6 had the highest sensitivity (95.1%) for ApHCM and the cut-off value was 0.185 mV. Considering ST-segment depression, the highest sensitivity (83.3%) for ApHCM was at lead V4 with the cut-off value of 0.05 mV. The summation of R wave in lead V5, neg T wave in lead V6 and ST-segment depression in lead V4 > 2.585 mV identified ApHCM with 90.2% sensitivity and 87.5% specificity, which was showed in Table 5. Representative ECGs of each group were shown in Fig. 4.

**Table 5:** Predictive values of electrocardiographic variables of the diagnosis of apical hypertrophic cardiomyopathy

	Cut off (mv)	Sensitivity	Specificity	PPV	NPV	Predictive accuracy
R wave in lead V5	2.07	90.2%	80.6%	72.5%	93.5%	92.3%
Neg T wave in lead V6	0.185	95.1%	61.1%	58.2%	95.7%	85.2%
Dep ST-segment in lead V4	0.05	83.3%	92.7%	76.5%	96.7%	82.0%
R wave in lead V5 + neg T wave in lead V6 + dep ST-segment in lead V4	2.585	90.2%	87.5%	80.4%	94.0%	94.1%

*Abbreviation*: *dep* Depressive, *neg* Negative

## Discussion

The current study revealed that the peak voltage of neg T and giant R clearly differed between ApHCM and NSTEACS, which occurred most frequently in leads V2-V6. We also showed that degree of ST-segment depression differed, particularly in leads V3-V6, between the two diseases. To our best knowledge, this is the first study to examine the change of ST-segment in patients with ApHCM and NSTEACS.

ApHCM has been recognized as Japanese-variant of hypertrophic cardiomyopathy since its high prevalence in the Japanese population [20, 21]. The etiology of ApHCM is multifactorial, with studies suggesting variants with a genetic predisposition and exclusive development during adulthood [22, 23]. ApHCM presents some particular electrocardiographic findings including deep inversion of the T waves (giant T waves) and the increase of the QRS complex voltage in the precordial leads [13]. However, in clinical setting, the ApHCM patients with giant neg T waves recorded in the ECG were always suspected of having acute coronary syndrome (ACS) [24]. Rogers reported a 61-year-old man admitted with chest pain, his ECG showed a biphasic T wave in lead V2, neg T waves in leads II and aVL, deep symmetrical T-wave inversions and ST depressions in leads V3–V6 [11]. He was suspected of ACS undergoing an emergent cardiac catheterization which revealed no coronary artery disease but a “spade like” pattern suggestive of ApHCM. Meghrajani reported a 66-year-old woman whose initial ECG showed T wave inversions in the lateral leads was diagnosed with type 2 myocardial infarction [25]. Coronary angiogram as well as cardiac left ventriculogram showed apical hypertrophy without coronary artery occlusion. From the ECG point of view, especially inverted T waves in V3-V6, ApHCM is often difficult to differentiate from NSTEACS. Previous studies had been confined to case reports or a relatively small number of patients. Herein we conducted an observational and retrospective study, the ECG findings could be conducive to differentiate ApHCM and NSTEACS early thus preclude the need for urgent coronary angiography and make accurate diagnosis and treatment essential for improved outcome.

To our knowledge, only the study reported by CHILLIK scrutinized ECG differences between ApHCM and NSTEMI [12]. They compared ECG changes between 19 patients with ApHCM and 19 patients with NSTEMI. They assessed neg T waves in leads V1-V6 showing a greater T-wave asymmetry. However, their study included only a small number of patients and they did not examine differences in ST-segment and the distributions or numbers of leads with neg T waves. Moreover, most previous studies showed T-wave typically displays > 10 mm inversions within the anterolateral leads in ApHCM, most prominent in V4 and V5 yet limb leads were received little attention. We therefore evaluated the R and T waves in all 12 leads. We identified the patients with ApHCM presented higher R and T wave voltage and peak voltage, similar to previous published studies. Besides, our study showed that ApHCM was associated with a greater ST-segment depression compared with NSTEACS. Meanwhile, the number of leads with neg T wave across 12-leads was more in patients with ApHCM. Giant neg T wave was exclusively found in ApHCM and the sum of R wave in lead V5, neg T wave in lead V6 and depressive (dep) ST-segment in lead V4 > 2.585 mV had the highest predictive value for ApHCM. Interestingly, we found on precordial leads of ApHCM, the amplitude of T-wave inversion displayed TV4 > TV5 > TV3, on the other hand, the amplitude of R-wave showed RV5 > RV4 > RV3. A series of new discoveries in our study would further facilitate differential diagnosis between ApHCM and NSTEACS.

The mechanisms responsible for the ECG differences and the underlying electrophysiologic conditions between ApHCM and NSTEACS are uncertain. These voltage criteria of ApHCM may be related to both LV hypertrophy and differences in localized wall thickness leading to disparities in the duration of repolarization. ApHCM is characterized by circular LV hypertrophy, while in NSTEACS, LV hypertrophy presented at the opposite side of the myocardium because of cardiac remodeling, thus it is not a circular hypertrophy [26]. In ApHCM, the mechanism for enormous R waves, dramatically in V3–V4 leads, is due to the apical distribution of hypertrophy opposite to the non-muscular elements of the fibrous cardiac skeleton of the mitral valve and annular plane. This result in an unopposed depolarization vectorial depolarization forces directed towards the cardiac apex [27]. Additionally, an alternative mechanism for prominent R waves is increased resistivity of cardiac muscle caused by fibrosis and myofibril disarray in the hypertrophied regions. Contrary to prominent R-waves, giant neg T waves isattributed to opposite vectorial orientation away from the cardiac apex. Neg T wave and dep ST-segment is considered a secondary phenomenon to high R-wave [26, 27].

Finally, it is important to apply these ECG rules into clinical context. Patient history is essential as ApHCM is a condition with varying clinical presentations. Active chest pain may suggest NSTEACS, whereas dyspnea usually imply ApHCM. In NSTEACS, you can find reciprocal ST changes or “mirror changes” on ECG, which is not usual in ApHCM [28]. Besides, ECG changes in ApHCM are generally stable against the rapid changes in ST-segment and T wave seen in serial ECGs of NSTEACS patients.

### Study limitations

Our study was performed as a retrospective analysis and at a single center. The number of patients, particularly ApHCM patients, was relatively small. Furthermore, we have to rule out the patients falling to meet our inclusion criteria (such as sinus rhythm). Therefore, our finding may not be available for the general group of patients with ApHCM or NSTEACS. Finally, the mean age of the enrolled ApHCM patients in our study was relatively high.

## Conclusions

Compared with NSTEACS, ApHCM patients presented higher voltage of R and neg T wave as well as greater ST-segment depression in the 12-lead ECG. Our proposed ECG characteristics can help to differentiate ApHCM from NSTEACS in clinical setting. Further studies in greater numbers of subjects are needed to verify our results.

## Data Availability

All data generated or analysed during this study are included in this published article.

## Abbreviations

A: Late diastolic inflow velocity
ACS: Acute coronary syndrome
ApHCM: Apical hypertrophic cardiomyopathy
AUC: Area under curve
Dep: Depressive
E: Early diastolic inflow velocity
ECG: Electrocardiogram
Em: Early diastolic annular tissue velocity
IVS: Interventricular septal
LAD: Left atrial diameter
LVEDd: Left ventricular end diastolic diameter
LVEDs: Left ventricular end systolic diameter
LVEF: Left ventricular ejection fraction
LVPW: Left ventricular posterior wall
neg T: Negative T
NSTEACS: Non-ST elevation acute coronary syndrome
NSTEMI: Non-ST segment elevation myocardial infarction
ROC: Receiver operator characteristic
UA: Unstable angina
